# The Relations of the Medical to the Legal Profession, Being the Introductory Address at the Opening of the Fifty-First Session of the College of Physicians and Surgeons, of New York

**Published:** 1857-02

**Authors:** 


*The Relations of the Medical to the Legal Profession, being the Introductory Address at the Opening of the Fifty-First Session of the College of Physicians and Surgeons, of New York.* By CHANDLER R. GILMAN, M.D. Prof. of Obstetrics and Medical Jurisprudence.

This address is mainly devoted to a discussion of the duties of medical men as witnesses in cases involving medico-legal questions. It is an interesting and valuable paper, and should be read by all who can procure a copy of it.

